# Enhancing Pediatric Pharmaceutical Care: Development and Evaluation of a Rectal Demonstration Model for Pediatric Patients with Inflammatory Bowel Diseases

**DOI:** 10.3390/children13081119

**Published:** 2026-08-21

**Authors:** Meike Ruschkowski, Gunter Flemming, Wieland Kiess, Astrid Bertsche, Thilo Bertsche, Martina Patrizia Neininger

**Affiliations:** 1 Drug Safety Center, Leipzig University and Leipzig University Hospital, 04103 Leipzig, Germany; meike.ruschkowski@uni-leipzig.de (M.R.); 2 Clinical Pharmacy, Institute of Pharmacy, Medical Faculty, Leipzig University, 04103 Leipzig, Germany; 3 Center for Pediatric Research, University Hospital for Children and Adolescents, Leipzig University Hospital, 04103 Leipzig, Germany; gunter.flemming@medizin.uni-leipzig.de (G.F.); wieland.kiess@medizin.uni-leipzig.de (W.K.); 4 Department of Neuropediatrics, University Medicine Greifswald, 17475 Greifswald, Germany; astrid.bertsche@uni-greifswald.de (A.B.); martina.neininger@uni-greifswald.de (M.P.N.); 5 German Center for Child and Adolescent Health (DZKJ), Partner Site Greifswald/Rostock, 17489 Greifswald, Germany

**Keywords:** demonstration model, handling errors in drug administration, rectal foam, inflammatory bowel disease, pediatric patients, adolescents

## Abstract

**Highlights:**

**What are the main findings?**

• A realistic rectal demonstration model was developed to visualize rectal foam administration and identify handling errors that are otherwise difficult to detect in routine pediatric care.
• Handling errors occurred frequently in adolescent patients; in 15/19 administrations, at least one high-risk error was observed, with releasing the pump dome too quickly being the most common error.

**What are the implications of the main findings?**

• The demonstration model can be integrated into pediatric routine care to identify and address clinically relevant handling errors during counseling.
• Training with the model may help adolescent patients practice correct rectal foam administration and thereby improve drug effectiveness and patient safety.

**Abstract:**

Background/Objectives: Inflammatory bowel disease (IBD) may require topical rectal treatment for distal colonic inflammation. Rectal foams or rectal enemas can reach deeper bowel sections but are complex to administer, especially for pediatric patients with IBD. Furthermore, it is a challenge to visualize this administration process in a structured manner. We aimed to design a rectal demonstration model to enable the visualization of rectal foam administration and to assess the clinical risk potentially associated with detectable errors. Methods: A rectal demonstration model was developed, handling errors were defined, and their clinical risk was rated. The model was tested for rectal foam administration by 19 adolescent patients (aged 12–18 years). Results: A total of 12 observable administration steps, including their clinical risk, were defined by an expert panel of physicians and pharmacists. Seven handling errors were rated with a high clinical risk, such as “Not shaking the spray can at all before administration”. In demonstrations by adolescent patients, a median of three (Q25/Q75; 2/4.5; min/max 1/7) handling errors were identified. The most frequent error was releasing the pump dome too quickly after pushing down (11/19, 58%). At least one error with high clinical risk (score: 5–6) was identified in 15/19 (79%) of patients’ administrations. Conclusions: The developed demonstration model facilitates the visualization of handling errors in rectal foam administration that would otherwise not be accessible in routine care. A high rate of clinically relevant handling errors was identified in adolescents. Prospectively, the model can be used to practice rectal foam administration and address patient handling errors to increase patient safety.

## 1. Introduction

Inflammatory bowel diseases (IBDs) such as Crohn’s disease and ulcerative colitis have a considerable incidence. For example, the incidence in adults is up to 15.9/100,000 person-years in the United States [1] and up to 74.0/100,000 in Europe [2], and the incidence for children and adolescents is up to 7.4/100,000 in the United States and up to 12.4/100,000 in some areas of Europe [3]. International guidelines recommend, among others, rectal dosage forms such as rectal foams for the treatment of IBDs as the first-line therapy, especially for distal ulcerative colitis [4,5,6,7,8,9]. For children and adolescents, suppositories are recommended as rectal therapy for mild to moderate IBDs, as data on rectal foam administration to children and adolescents are limited [5,10]. Rectal foams or rectal enemas reach deeper sections of the bowel, ensuring effectiveness by reaching the desired sites of action [11]. This may explain why gastroenterological specialists prescribe them off-label to pediatric patients. Nevertheless, it is already known from other complex dosage forms such as inhalation devices or rectal tubes that their administration is prone to handling errors [12,13,14,15,16]. Therefore, we hypothesized that rectal foam administration is also error-prone due to the complexity of this dosage form, especially if the pediatric patients administer the rectal foam themselves. In addition, rectal foam administration may be embarrassing for pediatric patients [17,18], so we assume that potential administration problems may not be addressed by the patients themselves. We also hypothesized that demonstration models could make those handling errors accessible, as this has been shown for other dosage forms [15,16,19]. However, existing rectal demonstration models, for example, for proctological examinations, are often not suitable to realistically simulate rectal drug administration.

Against this background, rectal foam administration represents a particular challenge for pediatric patients, especially when they are expected to self-administer the medication. Self-administration may be feasible from around 8 years of age, whereas younger children typically require assistance from their caregivers. Handling errors may remain unnoticed in routine care because the administration process is private, difficult to observe, and potentially embarrassing for patients to discuss. Therefore, our aim was to develop a realistic rectal demonstration model that allows pediatric patients to show how they administer rectal foams in a protected counseling setting. The model was intended to support the identification of individual handling difficulties, their frequency, and their potential clinical relevance. We explored the demonstration model under real-world conditions in pediatric patients with IBDs to assess whether it could be used in routine care.

## 2. Materials and Methods

### 2.1. Setting and Participants

After approval by the responsible ethics committee, we conducted an observational exploratory pilot study in the pediatric gastroenterological outpatient clinic of a university hospital in Saxony, Germany. We invited pediatric patients to participate in the study who were aged 8 to 18 years, had a diagnosis of IBD, and administered their rectal foam without parental support. This age range was chosen as children aged younger than 8 years usually do not administer the rectal foam themselves but receive help from their caregivers. Patients were required to have sufficient language skills, clinical constitution, and intellectual ability to perform the rectal foam administration on a demonstration model. Patients’ participation was voluntary, and written informed consent was obtained from all parents and participating patients aged ≥14 years. Data collection took place in a quiet environment to which unauthorized persons did not have access.

### 2.2. Development of the Rectal Model to Assess Errors in Drug Administration

#### 2.2.1. Characteristics of the Model

**Figure 1:**
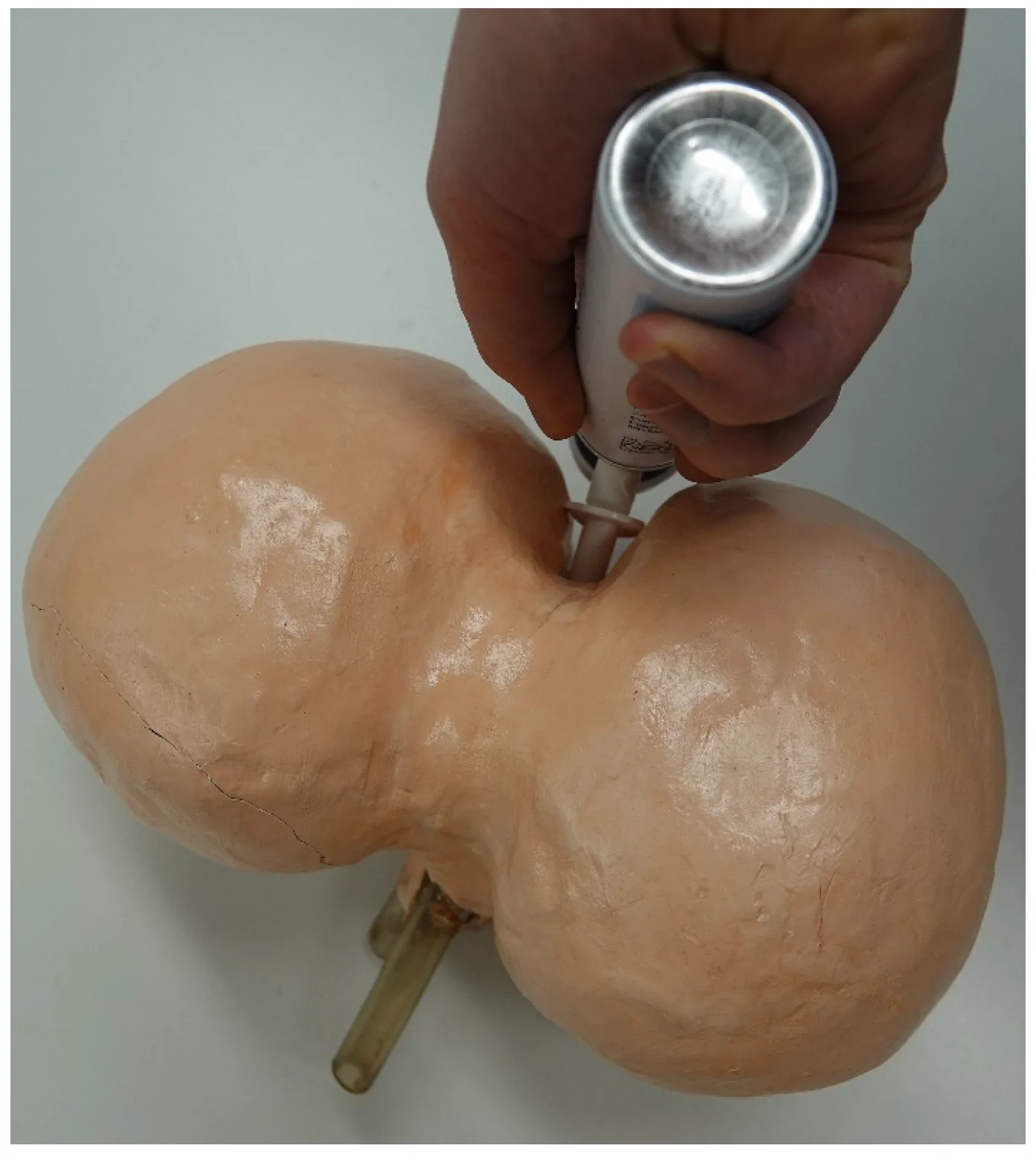
Newly developed rectal demonstration model. It represents a slightly forward-bent buttock, which corresponds to the correct body position to administer the rectal foam. Furthermore, it enables the visualization of how a rectal foam is administered. Small tubes lead to the front of the model so that it can be observed whether and how the administered drug emerges from the can. Pictures from each side of the model are shown in Figures S1–S3.

We designed a realistic rectal demonstration model for rectal foam administration, especially for pediatric patients. It represents a slightly bent-forward buttock, as rectal dosage forms are often administered in a standing or lying position (Figure 1). Small tubes lead to the front of the model so that it is possible to observe whether and how the administered drug comes out of the can and thus examine whether the pump dome was released correctly. The model was made from two cardboard tubes representing the legs and modeling clay in which two small tubes were embedded (rectal and vaginal opening); it was then painted with acrylic paint. The steps of the administration process were defined according to the Patient Information Leaflets (PILs) and Summaries of Product Characteristics (SmPCs) of rectal foams [20,21,22,23,24,25]. A structured observation sheet was developed to ensure consistent and standardized data collection during the demonstration process. Three clinical pharmacists tested whether each step could be reproduced with the demonstration model: Each of the pharmacists administered the rectal foam to the demonstration model while explaining what they were doing. Each procedure was documented using the developed observation sheet.

#### 2.2.2. Complementary Questionnaire to the Demonstration Model

As not all steps that are necessary for correct rectal foam administration are accessible with the demonstration model, such as toilet use, we also developed a short questionnaire to complement the demonstration model.

#### 2.2.3. Definition and Rating of Handling Errors in Drug Administration

Deviations from the process according to the PILs and SmPCs were classified as handling errors in drug administration. An expert panel consisting of three pediatricians from the gastroenterological team and four clinical pharmacists categorized the handling errors in rectal foam administration according to their potential clinical risk of causing harm to patients. The potential clinical risk was rated on a scale ranging from 1 (lowest risk) to 6 (highest risk), indicating the probability to which an error in drug administration potentially leads to a negative impact on effectiveness or on patient safety. The resulting clinical risk score reflects the median of the ratings of the experts.

### 2.3. Data Assessment

#### 2.3.1. Demonstration by Participants

The participating pediatric patients were asked to administer a rectal foam to the rectal demonstration model while a pharmacist from the study team observed. Along with the demonstration, they were asked to explain their actions in order not to miss anything. The observation and the participant’s explanations were documented on the observation sheet. In addition, the pharmacist documented the participant’s responses to the complementary questionnaire.

#### 2.3.2. Sociodemographic Data

We additionally collected the diagnosis of IBD, onset of the disease, current prescribed medication, and sociodemographic data from the medical records.

## 3. Results

### 3.1. Study Population

Of 23 patients who were treated with a rectal foam, a total of 19 adolescent patients (83%) agreed to show the administration of their prescribed rectal foam on a demonstration model. Reasons for declining study participation were autism spectrum disorder (one patient), high burden due to other studies (one patient), or no motivation (two patients). Most patients had already been administering rectal foams for a longer period before study participation; specifically, 18 patients (95%) had prior experience with rectal foam administration, and one patient had received a rectal foam prescription for the first time. Detailed patient characteristics are summarized in Table 1.

### 3.2. Development of the Rectal Model

In the development of the demonstration model, 12 observable administration steps were defined:

1. Push the applicator firmly onto the spout of the spray can;
2. Shake the spray can before administration;
3. Twist the dome on top of the spray can;
4. Turn the can upside down;
5. Hold the can with the pump dome pointing down during the entire period of administration;
6. Insert the applicator into the rectum;
7. Push the pump dome down fully after insertion;
8. Release the pump dome slowly after pushing down;
9. Take out the applicator from the rectum;
10. Remove the applicator from the can;
11. Dispose of the applicator in the plastic bag provided;
12. Turn back the pump dome.
  If an administration process requires two single doses, the number of administration steps increases to 15. For a second dose, the following steps are to be performed directly after step 8:
  - Leave the applicator in the rectum;
  - Push the pump dome down fully;
  - Release the pump dome slowly after pushing down.
  The errors in drug administration that occur if the steps are not carried out correctly were defined by the expert panel and are listed in Table 2. Pictures of each step are shown in Figures S4–S14.

### 3.3. Complementary Questionnaire to the Demonstration Model

In addition, four administration steps that are not accessible with the demonstration model were identified:

- Emptying the bowels before administration;
- Washing hands before and after the administration;
- Assuming correct body position for administration;
- Storing the rectal foam.

To assess those items, a complementary questionnaire was developed.

### 3.4. Clinical Relevance Rated by Expert Panel

The expert panel rated seven handling errors in drug administration with a high clinical risk (score: 5–6), e.g., “Dome on top of the spray can not twisted” (Table 2). Administration steps such as “Applicator not disposed of in the plastic bag provided” were rated with a low clinical risk (score: 1–2).

### 3.5. Types and Frequencies of Handling Errors in Rectal Foam Administration

Three (median; Q25/Q75; 2/4.5; min/max 1/7) handling errors were identified in the demonstrations of rectal foam administration by adolescent patients. The most frequent handling error observed in 11/19 (58%) patients was “Pump dome released too quickly after pushing down” (Table 2). This handling error belonged to the highest clinical risk category according to the expert panel. In 15/19 (79%) demonstration processes, at least one error with high clinical risk occurred (Table 2). In the complementary questionnaire, 18/19 (95%) adolescent patients reported emptying the bowels before rectal foam administration, and 1/19 (5%) reported emptying the bowels after the administration. Table 3 shows the answers given in the complementary questionnaire about emptying the bowels, washing hands, information about storage, and the body position.

## 4. Discussion

The developed rectal demonstration model enables the realistic visualization of rectal foam administration, facilitating the identification of handling errors that would otherwise not be visible in routine care. At least one handling error with high clinical risk was identified during drug administration by almost all adolescent patients. Because of its simple structure, the rectal demonstration model can be well integrated into routine care to identify errors associated with the administration of rectal dosage forms.

### 4.1. Benefits of the Demonstration Model

The developed realistic rectal demonstration model has been shown to be appropriate for visualizing rectal foam administration. An important difference compared with other already existing rectal models is that these models are mainly designed for examination purposes, for example, to palpate pathological findings such as colorectal tumors, and do not allow the administration of rectal dosage forms. In contrast, our newly developed demonstration model makes it possible to observe the administered drug emerging from the model, thereby verifying whether administration has been performed successfully. By means of a demonstration model, non-visible administration situations in routine care can be made accessible to healthcare professionals, caregivers, and patients. By using a demonstration model for the administration of drugs, error-prone steps can be identified, even if they are not perceived by the administering persons themselves [16]. Demonstration models have already been developed for other complex dosage forms to observe administration processes and detect errors in drug administration. To give some examples, the benefit of demonstration models has been shown for the administration of rectal and buccal emergency medication in pediatric patients with seizures, auto-injectors to be used in case of anaphylaxis, or to train medical professionals for emergencies [16,19,26]. The model we developed enables real-life observation of rectal foam administration and facilitates the identification of handling errors. The high frequency of handling errors underlines the high need for practical counseling. This is particularly relevant in adolescent patients, because they may experience rectal administration as embarrassing [17,18], and may not ask for support if they experience problems with their medication. Also, adolescents may experience their medication as intrusive or difficult to reconcile with their daily lives, for example, when treatment has to be planned around school activities, meeting up with friends, or school trips. Although the inclusion criteria allowed for pediatric patients aged 8 years and older, no eligible patients younger than 12 years received treatment with rectal foam. Consequently, the results of the model evaluation are based on participants aged 12 to 18 years, representing an adolescent cohort. Yet, the demonstration model may also be useful for counseling on rectal foam administration in younger children. For children whose rectal foam administration is primarily performed by caregivers, the model provides an opportunity to practice the procedure in a safe environment, thereby reducing potential concerns about causing harm to the child. The model is not limited to pediatric care and may be applicable to adult patients using rectal dosage forms. However, depending on the type and extent of the IBD, alternative dosage forms are also available for adults, resulting in a comparatively less prominent role for rectal foam [4,5,6,8,9]. However, this study focused on pediatric patients in whom rectal foam administration may be particularly challenging, while evidence on rectal foam administration remains limited.

### 4.2. Handling Errors in Rectal Foam Administration

In our study, at least one high-risk handling error was observed in four out of five adolescent patients during rectal foam administration. The most common error in more than 50% of demonstrations was releasing the pump dome too quickly, immediately after pushing down, although this must be done slowly to deliver the entire amount of drug into the patient’s body. Using our demonstration model, it was possible to visualize the amount of foam coming out, indicating incorrect drug administration, as the amount of foam was reduced compared to correct drug usage. This step would not be visible in routine care, which underlines the benefits of the model, as the direct consequences of incorrect drug administration become visible. Also, shaking the spray can was forgotten by more than 50% of participants, although this is an important step to ensure that the active ingredients and excipients are thoroughly mixed to obtain a stable foam with an even distribution of active ingredients.

### 4.3. Unobservable Steps in Rectal Foam Administration

Demonstration models have limitations as not all administration steps may be observable, because they are not realistically accessible. Yet by means of our designed complementary questionnaire, information about non-observable steps such as emptying the bowels before administration or body position for administration could be well assessed. To give an example, only 42% of the patients emptied the bowels before administration. However, emptying the bowels before administration is important to ensure drug effectiveness, because defecation may be triggered by the increased pressure on the rectal sphincter caused by the foam volume [27], and the drug also needs space to reach the correct site of action. Thus, the complementary questionnaire is an important means to identify error-prone process steps that should be addressed when patients are advised on the administration of rectal foams.

### 4.4. Benefits of Early Active Involvement of Pediatrics

Various studies showed the benefit of early active involvement of pediatric patients in their drug therapy and the associated drug administration [28,29]. For example, pediatric patients with IBDs who were counseled about their medications and associated adverse drug reactions were more adherent [30]. It has also been reported that knowledge about the medications used to treat IBDs resulted in children and, especially, adolescents having a better start to self-managing their disease and the associated management of drug therapy [29]. As our study showed that handling errors in rectal foam administration were frequent in adolescent patients, their involvement in the administration process should be encouraged at an early stage. This is particularly relevant because pediatric patients may not always accept prescribed therapy readily and may therefore refuse rectal administration because of shame, discomfort, or the perceived effort required for correct use [17,18]. In addition, rectal foam administration can interfere with everyday routines because patients need sufficient time, privacy, and the opportunity to remain lying down for a while after administration. Ideally, early involvement should be facilitated by using child-friendly materials such as the developed demonstration model. When developing our model, we therefore made sure that it is easy to reproduce and simple to use in order to facilitate straightforward integration into the counseling processes of pediatric patients in routine care.

## 5. Conclusions

Our exploratory study indicates that the developed rectal demonstration model of a buttock bent forward enables a realistic visualization of the complex process of rectal foam administration. The demonstration model is also useful for identifying handling errors in drug administration that are otherwise not accessible in routine care. Therefore, the demonstration model could also be useful for demonstrating correct drug handling and educating adolescent patients, thus contributing to increased drug safety. Future studies should focus on whether the use of the rectal demonstration model in patient training can lead to a reduction in handling errors in drug administration. In addition, future research could examine whether the demonstration model can be adapted to other rectal forms, such as suppositories or enemas, to support counseling beyond rectal foam administration. The model may also be useful for younger children, although counseling would primarily need to address the caregivers in this age group. Although the current model was deliberately designed to be analogue and readily reproducible, further technical development, such as the development of a 3D-printed demonstration model, could be considered.

## 6. Strengths and Limitations

We developed an easily replicable demonstration model for rectal foam administration that can be used to identify drug handling errors in pediatric routine care. Limitations of this study include that pediatric patients were included in only one outpatient clinic in Saxony, Germany. Nevertheless, it is a specialized center that covers a wide area of Saxony and neighboring federal states. Furthermore, all participants in our study were adolescents aged 12 years or older; therefore, no conclusions can be drawn for younger patients.

## Figures and Tables

**Table 1:** Patients’ characteristics.

Characteristics	Values
Patients	
Total number (male/female) [*n*]	19 (13/6)
Median age (Q25/75; min./max.) [years]	16.4 (15.4/16.9; 12.0/18.2)
Diagnosis [*n* (%)]	
Crohn’s disease	1 (5%)
Ulcerative colitis	13 (68%)
Inflammatory bowel disease unclassified/Indeterminate colitis	2 (10%)
Ulcerative pancolitis	2 (10%)
Not yet determined	1 (5%)
Median time since diagnosis (Q25/Q75; min/max) [months]	9.6 (4.8/21; 2.4/62.4)
Median number of prescribed drugs (Q25/Q75; min/max) [*n*]	3 (3/4; 2/5)

**Table 2:** Handling errors in the administration of rectal foam by adolescent patients [N = 19]. The predefined errors in drug administration are described in the order in which they were performed during the demonstration.

Defined Handling Errors in Drug Administration	Expert Rating of Clinical Risk of 1 to 6	Frequency of Observed Handling Errors in Drug Administration by Adolescent Patients [*n*/N (%)]
Applicator not firmly pushed onto the spout of the spray can	3	1/19 (5%)
Shaking the spray can before administration		
Not shaken at all	5	10/19 (53%)
Shaken less than 20 s (Salofalk^®^)/shaken less than 5–10 s (Claversal^®^)	3	5/19 (26%)
Dome on top of the spray can not twisted	6	1/19 (5%)
Spray can with pump dome pointing down not turned upside down	5	2/19 (11%)
Spray can with pump dome pointing down not held upside down during the entire time of administration	5	2/19 (11%)
Insertion of the applicator into the rectum		
Only halfway of the applicator	3	1/19 (5%)
Only the initial piece	4	0/19 (0%)
Pump dome not pushed down fully after insertion	6	1/19 (5%)
Pump dome released too quickly after pushing down	5	11/19 (58%)
Applicator was not left in the rectum *	5	1/10 (10%) **
Pump dome not pushed down fully after insertion *	6	0/10 (0%) **
Pump dome released too quickly after pushing down *	5	6/10 (60%) **
The applicator was not taken out from the rectum		
The applicator was taken out immediately after administration	5	6/19 (32%)
The applicator was taken out less than 10 s after administration	3	5/19 (26%)
Applicator not removed from the can	2	0/19 (0%)
Applicator not disposed of in the plastic bag provided	1	3/19 (16%)
Dome on top of the spray can not turned back	2	11/19 (58%)

* Only applicable in administrations requiring a second dose. ** Of the participating patients, 10 patients were required to administer a second dose according to their prescription.

**Table 3:** Complementary questionnaire to the demonstration [N = 19].

Question	Answers of Adolescent Patients [*n*/N (%)]
Do you always empty the bowels before administration? Answer options: Yes/No	Yes: 8/19 (42%)/No: 11/19 (58%)
Do you always wash your hands before the administration? Answer options: Yes/No	Yes: 13/19 (68%)/No: 6/19 (32%)
Do you always wash your hands after the administration? Answer options: Yes/No	Yes: 12/19 (63%)/No: 7/19 (37%)
In which body position do you administer the rectal foam? (Open question; multiple answers were possible)	
Lying on side with the lower leg stretched out and upper leg bent for balance {correct answer}	3/19 (16%)
Standing with one foot on a stool or chair {correct answer}	9/19 (47%)
Incorrect positions *.	7/19 (37%)
How do you store the rectal foam? (Open question; multiple answers were possible)	
Dark and locked storage place (e.g., cupboard, drawer) {correct answer}	15/19 (79%)
Bright and open storage place (e.g., shelf, windowsill) {incorrect answer}	3/19 (16%)
Refrigerator {incorrect answer}	1/19 (5%)

* Body positions such as “bending over”, “standing up”, “prone position” or “supine position”.

## Data Availability

The datasets generated during and/or analyzed during the current study are not publicly available due to the privacy protection requirements of the study participants but are available from the corresponding author on reasonable request.

## Supplementary Materials

The following supporting information can be downloaded at: https://www.mdpi.com/article/10.3390/children13081119/s1. Figure S1: Facing the rectum (and optional the vagina opening). Figure S2: Facing the side of the demonstration model. Figure S3: Facing the tubes (rectal and vaginal openings). Figure S4: Push the applicator firmly onto the spout of the spray can. Figure S5: Shake the spray can before administration. Figure S6: Twist the dome on top of the spray can. Figure S7: Turn can upside down. Figure S8: Hold can with pump dome pointing down during the entire period of administration. Figure S9: Insert the applicator into the rectum. Figure S10: Push the pump dome down fully after insertion. Figure S11: Release the pump dome slowly after pushing down. Figure S12: Take out the applicator from the rectum. Figure S13: Remove the applicator from the can. Figure S14: Dispose of the applicator in the plastic bag provided. Figure S15: Turn back pump dome.

## Abbreviations

The following abbreviations are used in this manuscript:

IBD	Inflammatory Bowel Disease
PIL	Patient Information Leaflet
SmPC	Summaries of Product Characteristics
