# Supplementary material for: Enhancing Pediatric Pharmaceutical Care: Development and Evaluation of a Rectal Demonstration Model for Pediatric Patients with Inflammatory Bowel Diseases
Source: Children (Basel). 2026 Aug 21;13(8):1119. doi: 10.3390/children13081119 (PMC13510760; doi:10.3390/children13081119)

Supplementary material

<u>Pictures from each side of the demonstration model</u>

Figure S1: Facing the rectum (and optional the vagina opening)

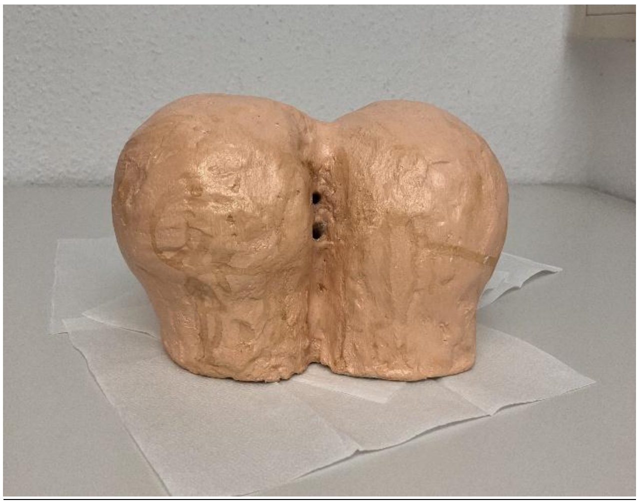

Figure S 2: Facing the side of the demonstration model

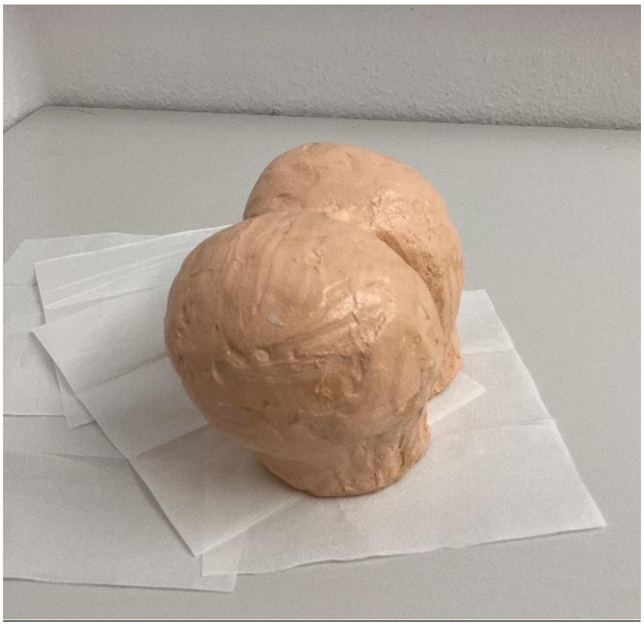

Figure S 3: Facing the tubes (rectal and vaginal openings)

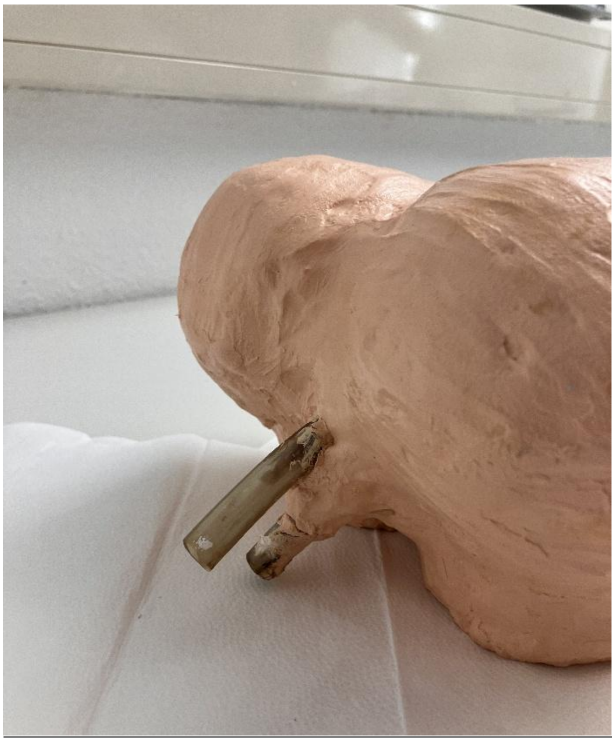

<u>Pictures illustrating the individual steps of the administration</u>

Figure S 4: Push the applicator firmly onto the spout of the spray can

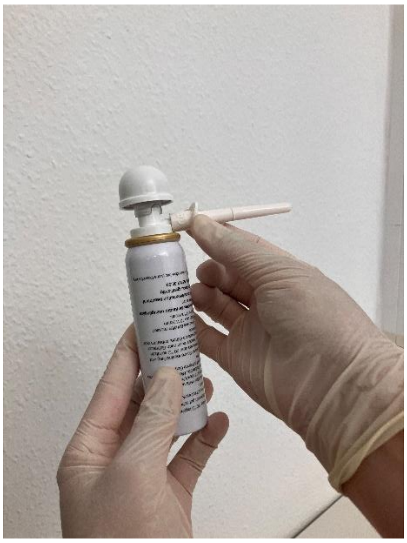

Figure S 5: Shake the spray can before administration

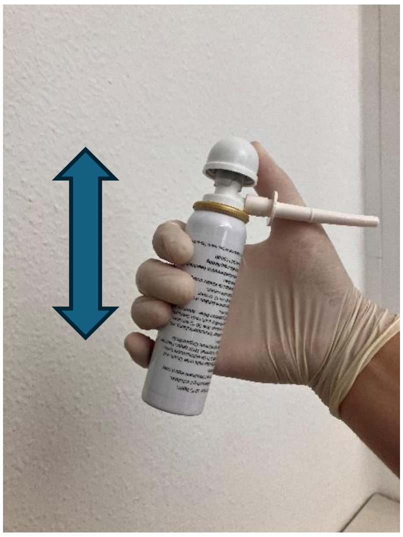

Figure S 6: Twist the dome on top of the spray can

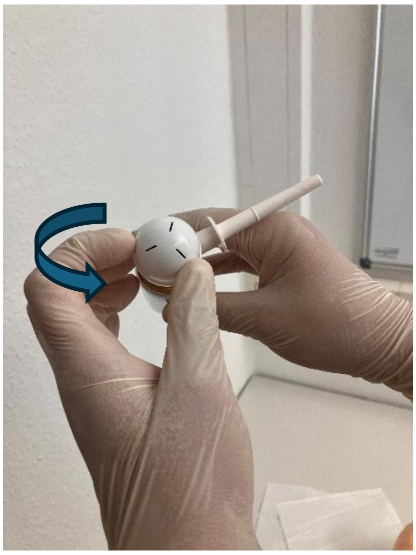

Figure S 7: Turn can upside down

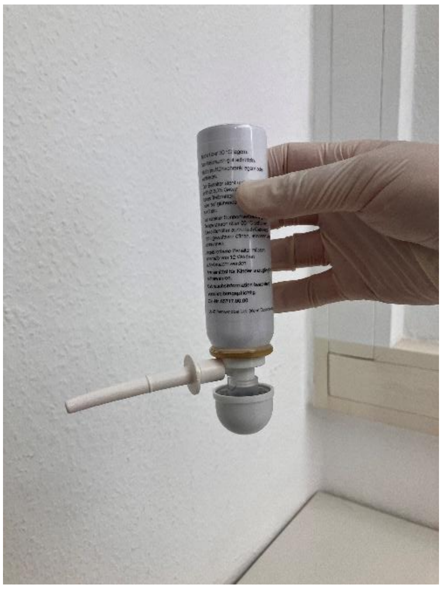

Figure S 8: Hold can with pump dome pointing down during the entire period of administration

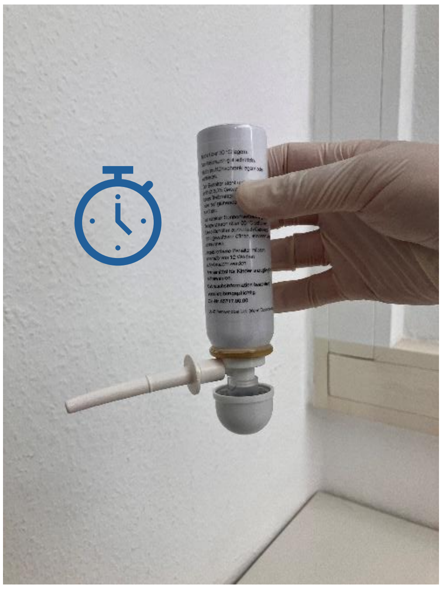

Figure S 9: Insert the applicator into the rectum

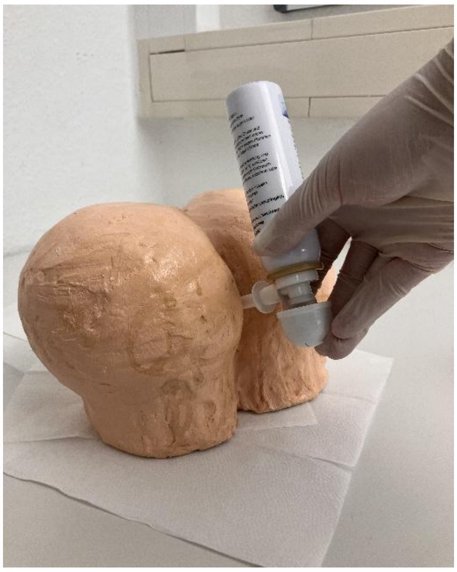

Figure S 10: Push the pump dome down fully after insertion

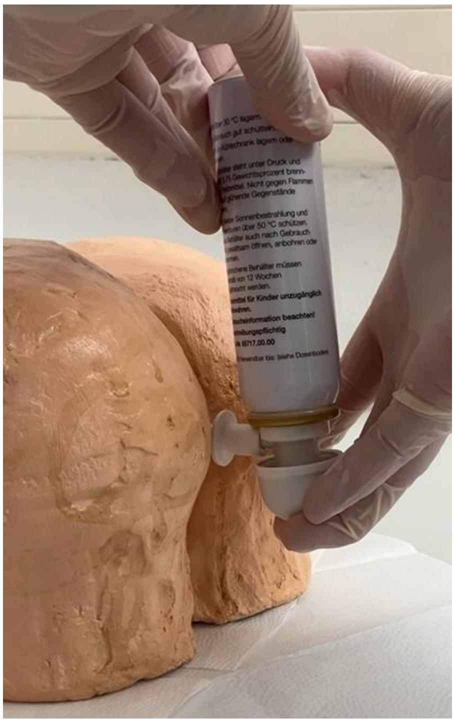

Figure S 11: Release the pump dome slowly after pushing down

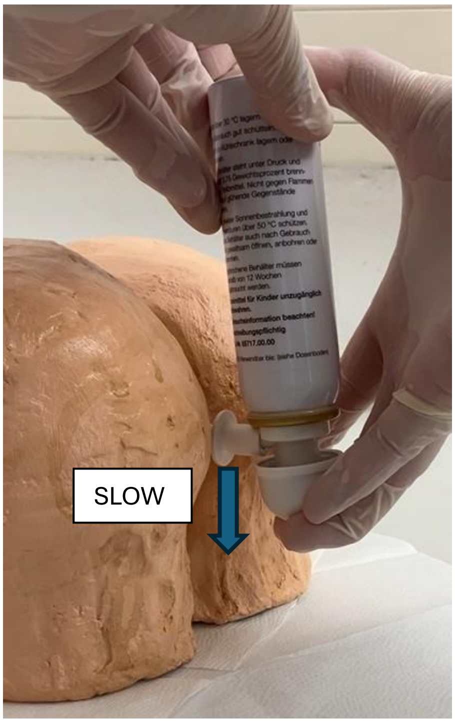

Figure S 12: Take out the applicator from the rectum

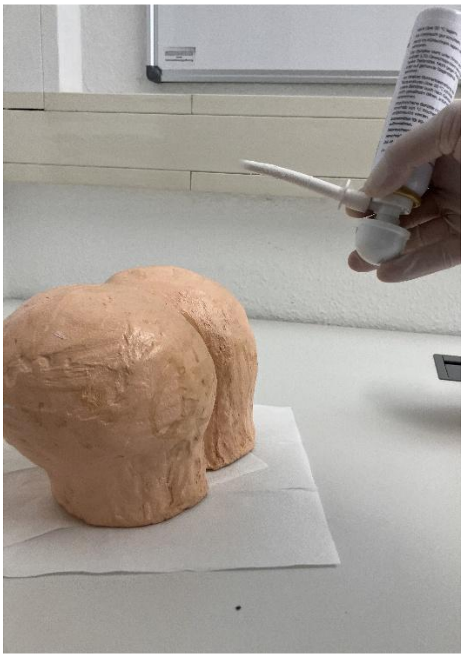

Figure S 13: Remove the applicator from the can

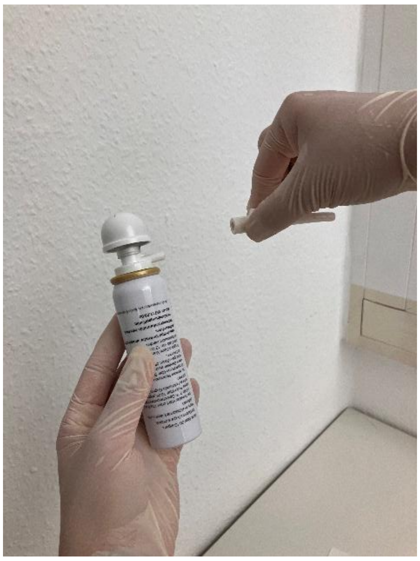

Figure S 14: Dispose of the applicator in the plastic bag provided

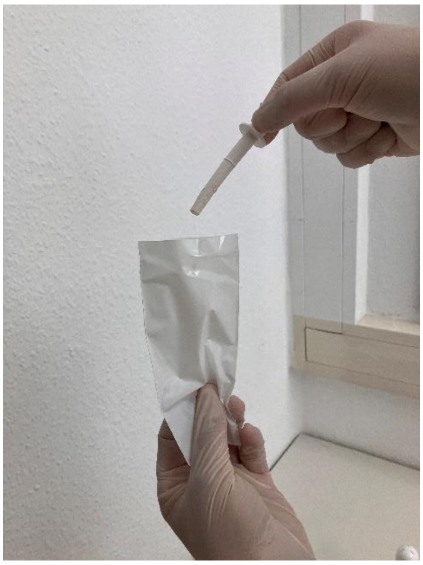

Figure S 15: Turn back pump dome

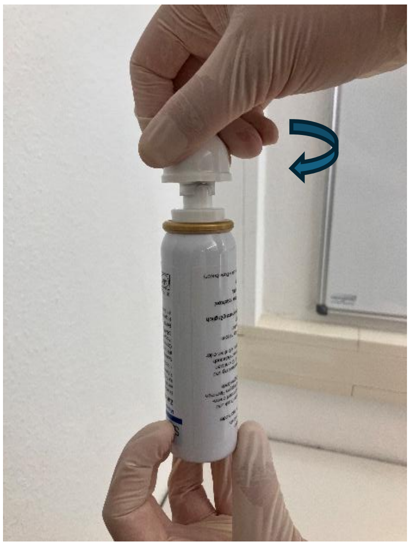

Supplement: Supplementary file 1 [file children-13-01119-s001.zip › children-4475795-supplementary.pdf]
